# Safety of Drugs Used in Difficult-to-Treat Epileptic Syndromes: A Disproportionality Analysis Using the Eudravigilance Database

**DOI:** 10.3390/ph18121895

**Published:** 2025-12-16

**Authors:** Arianna Scala, Teresa Angela Trunfio, Chiara Pennisi, Giovanni Enrico Lombardo, Vincenzo Micale, Serena Di Martino, Giorgia Fiorenza, Adriana Carol Eleonora Graziano, Marilena Briglia, Fabio Allia, Giovanni Giurdanella, Roberta Malaguarnera, Rosalia Battaglia, Cecilia Gozzo, Fanny Erika Palumbo, Calogero Vetro, Giovanni Improta, Mario Damiano Toro, Filippo Drago, Giovanni Luca Romano, Lucia Gozzo

**Affiliations:** 1 Department of Public Health, University of Naples Federico II, 80131 Naples, Italy; ariannascala7@gmail.com (A.S.); teresa.trunfio@gmail.com (T.A.T.); giovanni.improta@unina.it (G.I.); mariodamiano.toro@unina.it (M.D.T.); 2 Department of Medicine and Surgery, University of Enna “Kore”, 94100 Enna, Italy; chiara.pennisi@unikore.it (C.P.); giorgia.f1d@gmail.com (G.F.); adriana.graziano@unikore.it (A.C.E.G.); marilena.briglia@unikore.it (M.B.); fabio.allia@unikore.it (F.A.); giovanni.giurdanella@unikore.it (G.G.); roberta.malaguarnera@unikore.it (R.M.); rosalia.battaglia@unikore.it (R.B.); giovanniluca.romano@unikore.it (G.L.R.); 3 Department of Biomedical and Biotechnological Sciences, School of Medicine, University of Catania, 95123 Catania, Italy; vmicale@unict.it (V.M.); serena.dm.92@gmail.com (S.D.M.); prof.filippodrago@gmail.com (F.D.); 4 Radiology Unit, Humanitas Istituto Clinico Catanese, 95045 Catania, Italy; ceciliagozzo91@gmail.com; 5 Division of Hematology, A.O.U. Policlinico “G.Rodolico—S. Marco”, 95125 Catania, Italy; fannypalumbo@gmail.com; 6 Hematology and Bone Marrow Transplantation Unit, Hospital of Bolzano (SABES-Azienda Sanitaria dell’Alto Adige), Teaching Hospital of Paracelsus Medical University, 39100 Bolzano, Italy; gerovetro@gmail.com; 7 Department of Special Surgery Ophthalmology Department, University of Jordan, Amman 11942, Jordan; 8 Clinical Trial Unit, University Hospital of Catania, 95125 Catania, Italy

**Keywords:** epileptic syndromes, stiripentol, cannabidiol, fenfluramine, adverse events, eudravigilance, disproportionality analysis

## Abstract

**Background/Objectives**: Difficult-to-treat epileptic syndromes include conditions typically emerging in the first years of life and are characterized by a high rate of drug refractoriness. This study aimed to better define the safety profile of drugs used as adjunctive therapies for seizures associated with these syndromes using real-world pharmacovigilance data. **Methods**: We retrospectively analyzed the publicly available data regarding Individual Case Safety Reports (ICSRs), presenting stiripentol, cannabidiol, or fenfluramine as suspected drugs, reported on the Eudravigilance database until the third quarter of 2024. Data were evaluated with descriptive analyses and then with disproportionality measures, including the reporting odds ratio. **Results**: A total of 5986 ICSRs met the inclusion criteria (71.6% from cannabidiol, 14.5% fenfluramine, and 13.9% stiripentol). Significantly higher probabilities of reporting Cardiac disorders, Vascular disorders, and Respiratory, thoracic, and mediastinal disorders were observed with fenfluramine. Cannabidiol was associated with Product issues, whereas stiripentol was associated with injury, poisoning, procedural complications, Metabolism and nutrition disorders, and Blood and lymphatic system disorders. **Conclusions**: Our analysis did not highlight new and unexpected serious safety signals but confirmed the need to strictly monitor patients for the risk of adverse events. However, further prospective studies are required to better clarify the safety profile of these drugs in order to optimize their use.

## 1. Introduction

Epileptic encephalopathies are severe forms of epilepsy which typically occur early in infancy and result in reduced cognitive function [1,2,3,4]. These disorders can be part of epileptic syndromes and can be characterized by generalized or recurrent focal seizures and being severe and usually refractory to standard antiepileptic drugs (AEDs), including benzodiazepines, phenobarbital, sodium valproate, lamotrigine, and topiramate.

Dravet syndrome (DS) and Lennox–Gastaut syndrome (LGS) are two rare but very severe epileptic syndromes [5]. DS, also known as severe myoclonic epilepsy of infancy (SMEI), is a genetic condition caused frequently by the loss-of-function of *SCN1A* variants, which typically develops in the first year of a life with frequent, prolonged seizures commonly triggered by hyperthermia [6,7,8]. LGS is a severe form of epilepsy, characterized by multiple types of seizures (tonic axial, atonic, absence, myoclonic, and generalized tonic–clonic seizures) and intellectual disability [9]. It can be associated with encephalitis, meningitis, tuberous sclerosis, brain malformations, and brain injury. Even in this case, treatment with AEDs is often ineffective.

Patients with DS and LGS are usually in polypharmacy, treated with a median of three AEDs [10,11,12,13,14,15].

The use of these drugs should be optimized to reduce the burden of seizures, as well as to minimize adverse events (AEs).

Valproate (VPA) is recognized as the first-line medication [11,16]; for subsequent lines, various AEDs have been approved as adjunctive therapies.

Among the pharmacological agents approved in the European Union as adjunctive therapy for the management of pediatric epilepsy-related seizures, in particular for DS, we can find stiripentol, cannabidiol, and fenfluramine.

In 2007, stiripentol (Diacomit^®^) received approval in Europe as an orphan drug for managing bilateral tonic–clonic seizures (BTCS) in children with DS [17]. It is intended for use as an add-on therapy alongside clobazam (CLB) and VPA, in cases where these drugs are not sufficient to control seizures [18,19]. Several mechanisms of action have been proposed for stiripentol, including enhanced GABAergic neurotransmission, inhibition of lactate dehydrogenase, reduction in calcium-mediated toxicity in hippocampal neurons with NMDA receptors, and the inhibition of calcium channels [20]. Several studies have highlighted that stiripentol is a generally well-tolerated and effective medication for reducing the frequency and duration of epileptic seizures [21,22], with certain AEs occurring more frequently, such as weight loss, decreased appetite, and drowsiness [23,24].

Cannabidiol, along with Δ9-tetrahydrocannabinol (THC), is one of the bioactive compounds found in the *Cannabis* plant [25]. Unlike THC, cannabidiol is not a psychoactive substance and exhibits anti-inflammatory, neuroprotective, and antioxidant properties [26]. Although the exact anticonvulsant mechanism of action has not yet been fully elucidated, cannabidiol has proven effective in suppressing seizures in animal models of epilepsy [26]. It has been proposed that cannabidiol may modulate neuronal excitability by interacting with various targets involved in the functional regulation of neuronal excitability, such as the transient receptor potential vanilloid 1 (TRPV1), the equilibrative nucleoside transporter 1 (ENT1), the orphan G protein-coupled receptor 55 (GPR55) [27], the voltage-gated sodium channels [28], the dopamine D2/D3 receptors [29,30], and the cannabinoid CB1 receptors [31,32,33]. Cannabidiol has been approved in the European Union under the name Epidyolex^®^ (GW Pharma [International] B.V.), in combination with CLB, for the treatment of seizures associated with LGS and DS in patients aged two years and older [34]. Additionally, in Europe, cannabidiol is authorized as an adjunct therapy for seizures associated with the tuberous sclerosis complex (TSC) in patients over the age of two [35]. The primary AEs associated with cannabidiol use include drowsiness, diarrhea, vomiting, and pyrexia [36,37,38,39].

Fenfluramine (Fintepla^®^) is an anticonvulsant drug with a dual mechanism of action, functioning both as an agonist of the serotonergic system and a positive allosteric modulator of sigma-1 receptors (σ1-Rs) [40]. It has been suggested that its interaction with serotonin (5-HT) receptors enhances inhibitory transmission mediated by γ-aminobutyric acid (GABA), while the activation of σ1-Rs is predominantly associated with a reduction in excitatory glutamatergic signaling [40]. Previously, the drug was used at high doses as an appetite suppressant in adults with obesity, but its marketing was discontinued due to an increased risk of serious cardiovascular events, valvular heart disease (VHD), and pulmonary arterial hypertension (PAH). More recently, it has been approved in Europe at significantly lower doses for the treatment of seizures associated with DS and LGS, as an adjunctive therapy to other anticonvulsants in patients older than 2 years [41]. Fenfluramine exhibits a favorable tolerability profile, with the most commonly reported AEs across multiple studies being reduced appetite, fatigue, and somnolence [40,42,43].

Although the tolerability of these drugs has been assessed in several clinical studies, post-marketing surveillance is essential to ensure the safe use of these medications. Indeed, post-marketing studies on pharmacovigilance databases potentially enable the identification of rare or severe AEs, which may not have emerged during the drug development phase due to time constraints or limited study populations [44].

The aim of this study was to analyze data from the Eudravigilance database to comprehensively assess the safety profile of these drugs.

## 2. Results

Our analysis showed a high prevalence of ICSRs from cannabidiol, followed by fenfluramine and stiripentol. Significantly higher probabilities of reporting Cardiac disorders, Vascular disorders, and Respiratory, thoracic, and mediastinal disorders were observed with fenfluramine. Cannabidiol was associated with Product issues, whereas stiripentol was associated with Injury, poisoning, procedural complications, Metabolism and nutrition disorders, and Blood and lymphatic system disorders.

**Figure 1:**
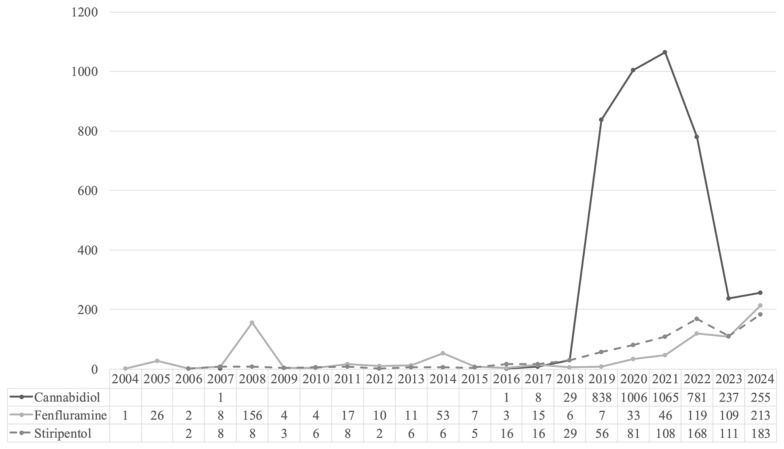
Temporal distribution of Individual Case Safety Reports (ICSRs) related to stiripentol, cannabidiol, and fenfluramine.

Overall, 5896 ICSRs related to stiripentol (*n* = 822; 13.9%), cannabidiol (*n* = 4222; 71.6%), and fenfluramine (*n* = 852; 14.5%) were retrieved from the Eudravigilance database for the reference period (Figure 1). The number of cases reported increased year by year, with the highest number of reports observed in 2021 for cannabidiol (*n* = 1065; 25.2%), and in 2024 for fenfluramine (*n* = 213; 25%) and stiripentol (*n* = 183; 22.3%).

Table 1 summarizes the main characteristics of the ICSRs for each drug.

The incidence of events in males accounted for a larger proportion than females, except for fenfluramine (stiripentol *n* = 423; 51.46%; cannabidiol *n* = 2149; 50.9%; fenfluramine *n* = 243; 28.52%; *p* < 0.001). Adult patients (≥18 years old) were more represented than the pediatric population (<18 years old), except for stiripentol.

In terms of seriousness, more than 80% of ICSRs indicated at least one ADR classifiable as serious, although less frequently in the fenfluramine group (stiripentol *n* = 728, 88.6%; cannabidiol *n* = 3619, 85.7%; fenfluramine *n* = 713, 83.7%; *p* = 0.015). The outcome recovered/resolved was reported for 2043 cases, more frequently for cannabidiol (*n* = 1423, 16.1%; *p* = 0.009), whereas a fatal event was reported for 612 cases, more frequently for cannabidiol (*n* = 503, 5.7%; *p* = 0.001). However, outcome data were available in less than 50% of ICSRs (stiripentol *n* = 620, 27.9%; cannabidiol *n* = 3566, 40.4%; fenfluramine *n* = 814, 39.1%).

More than 80% of ADRs were reported by healthcare professionals, apart from stiripentol (stiripentol *n* = 444, 54.01%; cannabidiol *n* = 3974, 94.13%; fenfluramine *n* = 692, 81.22%). Unfortunately, further information about the sender is not available. ICSRs are reported according to the sender, identified only as a healthcare or non-healthcare professional.

The largest number of AEs related to stiripentol and cannabidiol came from France (8.03% and 9.85, respectively), whereas in Germany, ICSRs associated with fenfluramine were reported more frequently (15.49%).

“Nervous system disorders” was the most represented reaction group (stiripentol *n* = 563; 26.6%; cannabidiol *n* = 2292; 27.1%; fenfluramine *n* = 368; 19.1%; Table 2).

The most frequent suspected reactions related to this SOC were ‘seizure’ (stiripentol *n* = 423; cannabidiol *n* = 1609; fenfluramine *n* = 151) and ‘somnolence’ (stiripentol *n* = 84; cannabidiol *n* = 207; fenfluramine *n* = 32) (Table 3).

Significantly disproportionately, reporting for fenfluramine-related reports compared to the other drugs was observed for “Cardiac disorders” (ROR = 26.29; 95% CI 25.422–27.187), “Vascular disorders” (ROR = 5.271; 4.959–5.602), and “Respiratory, thoracic, and mediastinal disorders” (ROR = 4.678; 4.579–4.779) (Table 4; Tables S1–S4). The most frequently suspected reactions related to these SOCs were pulmonary hypertension (‘pulmonary arterial hypertension’, PAH, *n* = 27, and ‘pulmonary hypertension’ *n* = 62) and valvular heart disease (VHD), including ‘aortic valve incompetence’ (*n* = 79), ‘cardiac valve disease’ (*n* = 51), ‘mitral valve incompetence’ (*n* = 86) ‘mitral valve stenosis’ (*n* = 11), and ‘tricuspid valve incompetence’ (*n* = 44; Table 5). Moreover, significantly higher probabilities of reporting were observed for “Product issues” with cannabidiol (ROR = 5.989; 5.148–6.968), and “Injury, poisoning, and procedural complications” (ROR = 3.033; 2.998–3.068), “Metabolism and nutrition disorders” (ROR = 2.867; 22.796–2.94), and “Blood and lymphatic system disorders” (ROR = 2.64; 2.366–2.947) with stiripentol (Table 6 and Table 7).

## 3. Discussion

A lot of AEDs can be used to control seizures in epileptic syndromes in the first or subsequent lines (such as clobazam or valproate). We focused our analysis on drugs with similar clinical applications in the context of pediatric epilepsy-related seizures. Stiripentol, cannabidiol, and fenfluramine are AEDs with different mechanisms of action that have been approved for specific difficult-to-treat epileptic syndromes. We found differences in terms of incidence according to age and gender, but we cannot draw final conclusions due to the lack of detailed information on the use of these drugs. In particular, adults were more represented than pediatric patients for fenfluramine and cannabidiol. These drugs are approved for epileptic syndromes, but their use is not limited to children and even adults or the elderly can also be treated according to the summary of the products’ characteristics. Indeed, these syndromes generally develop in the first years of life and can persist into adulthood, though seizure types and frequency may change [45,46].

The safety profile of these drugs is quite similar, including the occurrence of some common AEs such as weight loss, decreased appetite, and drowsiness/somnolence. Nevertheless, clinical trials and post-marketing surveillance allowed us to recognize specific ADRs, such as neutropenia, thrombocytopenia, nausea, and vomiting for stiripentol, anemia, diarrhea, and infections for cannabidiol, and gastrointestinal and cardiovascular disorders for fenfluramine.

Many of the above ADRs are often due to the other anticonvulsants used in combination (e.g., clobazam and valproate) and may resolve with dose reduction.

Our analysis showed increased reporting frequencies of “Nervous system disorders” for the three drugs. The most frequent events were ‘seizure’ and similar reactions (‘atonic seizure’, ‘epilepsy’, ‘generalized tonic–clonic seizure’, ‘myoclonic epilepsy’, ‘partial seizure’, ‘petit mal epilepsy’, ‘seizure cluster’, ‘status epilepticus’, and ‘tonic convulsion’). Most of these ADRs can be derived from insufficient therapeutic control of pre-existing diseases rather than from exposure to the drug. However, seizure aggravation (or the development of new types of seizures) can occur, in theory, with all AEDs [47]. The mechanisms of this paradoxical effect are mostly unknown and may be related to specific pharmacodynamic mechanisms, e.g., enhanced GABA transmission or the blockade of voltage-gated sodium channels [48].

The SmPC of fenfluramine describes a possible clinically relevant increase in seizure frequency, which may occur during treatment and may require dose adjustment or discontinuation, as with other AEDs [41]. Indeed, seizure was among the most frequent AEs leading to study withdrawal in clinical trials and status epilepticus was observed in three patients (3%) treated with the highest dose of fenfluramine [49].

Even for cannabidiol, a clinically relevant increase in seizure frequency may occur during treatment, but the frequency of status epilepticus in the phase 3 clinical trials was similar to that of the placebo [35].

To the best of our knowledge, stiripentol seems to not be associated with paradoxical seizures [17], and the reaction ‘seizure’ is not reported in its SmPC. Therefore, our findings should be further explored to figure out the link between the events that are more frequently reported on the Eudravigilance database and treatment with stiripentol.

As expected, ‘somnolence’ was the second most reported neurological event, being already recognized as a ‘very common’ ADR in the three SmPCs.

According to the disproportionality analysis, the most significant signals at the SOC level were “Cardiac disorders”, “Vascular disorders”, and “Respiratory, thoracic, and mediastinal disorders” for fenfluramine compared to the other drugs.

These results are in accordance with the known safety profile of fenfluramine used as an anorectic agent. The drug was approved for adults to reduce body weight, but it was withdrawn in 1997 based on the increased risk of VHD and PAH, even if most of the cardiopulmonary disorders improved following drug discontinuation [50,51]. In these patients, fenfluramine was prescribed at higher dosages (>40–60 mg daily or 0.5–2.1 mg/kg/day) than those approved for epileptic syndromes, ranging from 0.2 mg/kg/day–0.7 mg/kg/day, with a maximal recommended daily dose of 17–26 mg [48,52,53,54,55,56,57,58,59,60,61,62]. No cases of VHD or PAH were observed during the studies for epileptic syndromes. These results have been confirmed in long-term studies in patients treated for 3 years. These data support the cardiovascular safety of fenfluramine at lower dosages used for seizures compared to the higher dosages used for obesity [49,63,64]. However, post-marketing data showed that VHD and PAH can also occur with low doses of fenfluramine and cardiac monitoring using echocardiography is recommended prior to starting treatment to exclude any pre-existing diseases: every 6 months for the first 2 years and annually thereafter [41]. In the case of pathological abnormalities on the echocardiogram, it is recommended to evaluate the benefit of continuing treatment compared to the risk of ADRs.

To confirm that these ADRs were related to the use of fenfluramine for epilepsy, we selected the ICSRs from the year of approval for epileptic syndromes (2020), and we found a considerable reduction in the number of events (Table 5). However, this does not exclude the risk of VHD and PAH and patients should continue to be monitored.

The pathophysiology of these side effects is still under debate but may be linked to the interaction with serotonin receptors and the consequent growth of pulmonary smooth muscle cells [65,66]. Moreover, 5HT2B receptors can be specifically involved with the hyperplasia responsible for VHD [67].

Currently, these events are listed in the SmPC of fenfluramine with a frequency ‘not known’ [41], and further investigations are thus needed to define the burden of this association.

Significantly higher probabilities of reporting “Product issues” were observed with cannabidiol, in particular ‘product supply issue’ and ‘product distribution issue’. These events were observed only with cannabidiol and were reported for more than 85% of countries from the Non-European Economic Area (Table S5). As far as we know, these issues have not been raised previously, and we can only speculate that they may be related to the nature of the product as a cannabis-derived drug.

Finally, we found a significant association for stiripentol with the onset of “Blood and lymphatic system disorders”, “Injury, poisoning, and procedural complications”, and “Metabolism and nutrition disorders”.

Neutropenia is a common AE associated with the administration of stiripentol, and blood counts should be assessed prior to starting treatment, and then monitored every 6 months [17]. Instead, thrombocytopenia has been recognized as a rare event.

As regards to “Metabolism and nutrition disorders”, it is well known that decreased appetite represents an expected event in patients treated with stiripentol, but also with fenfluramine and cannabidiol, as reported in their SmPCs [17,35,41]. This represents a critical point, especially for the pediatric population, which may experience growth retardation given the frequency of gastrointestinal ADRs (nausea and vomiting). Therefore, the growth rate of children under these treatments should be carefully monitored.

Finally, ‘product use in unapproved indication’ (e.g., LGS, frontal lobe epilepsy, epileptic encephalopathy, partial seizures, febrile convulsion, and idiopathic generalized epilepsy) was the most reported ADR in the group “Injury, poisoning, and procedural complications”, supported by data emerging regarding off-label use in other forms of epilepsy. Indeed, stiripentol demonstrated to be effective in different seizure types for pediatric patients with drug-resistant epilepsy, apart from DS [18,68,69,70,71,72,73,74,75].

Although our findings provide a comprehensive perspective in the evaluation of ADRs related to the drugs under evaluation, the results of the present study should be observed in the light of some limitations, including the lack of detailed information about the treatment, the number of patients effectively treated in the reference period, the specific patients’ characteristics, the presence of multiple suspected drugs in ICSRs, the risk of under-reporting compared to the global clinical population, and the difficulty in identifying confounders. Indeed, at this access level, detailed information about drug use is not available. In particular, treatment duration was available only for a small percentage of ICSRs (8–13%). Therefore, it is difficult to conduct further analyses or construct hypotheses. Moreover, even when this information is available, is not possible to figure out if the treatment began in pediatric age, because we only have the treatment duration and the onset age of ADRs., e.g., for an ICSR, we know that the patient experienced the ADR at the age 18–64 years and that cannabidiol was used for 500 days, but we do not know the exact age and when cannabidiol was started.

## 4. Materials and Methods

### 4.1. Data Source and Study Design

This pharmacovigilance study was conducted by analyzing AE reports from the Eudravigilance database, from December 2001 until the third quarter of 2024. Data on Individual Case Safety Reports (ICSRs) presenting the suspected drugs of stiripentol, cannabidiol, or fenfluramine were retrieved using the publicly available Eudravigilance access platform (www.adrreports.eu). The data access level used for our analysis was the one indicated as “Stakeholder Group II: Healthcare professionals, patients and the general public” in the Eudravigilance access policy.

Suspected adverse drug reactions (ADRs) were grouped according to the Medical Dictionary for Regulatory Activities (MedDRA^®^) [75,76,77,78,79,80] and defined as serious if they were fatal, life-threatening, required hospitalization or prolonged existing hospitalization, resulted in persistent or significant disability, represented a congenital anomaly/birth defect, or other medically important condition [81,82].

For the outcomes, ADRs were classified using the standardized terminology: ‘recovered/resolved’, ‘recovering/resolving’, ‘recovered/resolved with sequelae’, ‘not recovered/not resolved’, ‘fatal’, and ‘unknown’, on the basis of what was reported in the ICSR.

The ADR expectedness was verified by consulting the Summary of Product Characteristics (SmPCs) available in the European Medicines Agency (EMA) database [17,35,41].

### 4.2. Data Analysis

Starting with an Excel spreadsheet (Microsoft Office, Excel, Microsoft Corporation, Redmond, Washington) where all variables of interest were collected, the analysis was performed by developing a special script using the open-source software RStudio, version 2025.05.0-496 [83]. Descriptive statistics were used to summarize data, reporting frequencies and percentages for categorical data and median values for continuous data.

In particular, descriptive statistics were implemented to represent how the three samples were distributed according to the different variables (such as demographic, reporting, or outcome) collected. In order to assess the differences between the active ingredients, the proportion test, mathematically equivalent to the chi-square test, was conducted [84]. The associations between the drugs and AEs were determined by the reporting odds ratio (ROR), the proportional reporting ratio (PRR), the Bayesian confidence propagation neural network (BCPNN), and the multi-item gamma Poisson shrinker (MGPS) algorithms, which were based on the disproportionality analysis (Table 8).

Various tables were obtained by isolating one active ingredient from the others and for the different system organ classes (SOCs). On these contingency tables, the formulae for calculating the signal detection methods were implemented using references from the literature [85]. For each, 95% confidence intervals were given, and the thresholds reported in Table 9 were applied to them.

## 5. Conclusions

To the best of our knowledge, this is one of the first pharmacovigilance studies to compare the safety and tolerability profiles of AEDs used in difficult-to-treat epileptic syndromes.

Our analysis did not reveal new and unexpected serious safety signals but confirmed the need to strictly monitor patients to optimize treatment and minimize AE onset.

## Figures and Tables

**Table 1:** Characteristics of Individual Case Safety Reports (ICSRs).

	Stiripentol	Cannabidiol	Fenfluramine
**Age group**	822 (100)	4222 (100)	852 (100)
Not Specified	226 (27.49)	581 (13.76)	216 (25.35)
0–1 Month	0 (0)	2 (0.05)	2 (0.23)
2 Months–2 Years	94 (11.44)	157 (3.72)	28 (3.29)
3–11 Years	262 (31.87)	991 (23.47)	130 (15.26)
12–17 Years	113 (13.75)	661 (15.66)	92 (10.8)
18–64 Years	126 (15.33)	1732 (41.02)	356 (41.78)
65–85 Years	1 (0.12)	93 (2.2)	27 (3.17)
More than 85 Years	0 (0)	5 (0.12)	1 (0.12)
**Sex**	822 (100)	4222 (100)	852 (100)
Female	383 (46.59)	1948 (46.14)	506 (59.39)
Male	423 (51.46)	2149 (50.9)	243 (28.52)
Not Specified	16 (1.95)	125 (2.96)	103 (12.09)
**Reporter group**	822 (100)	4222 (100)	
Healthcare Professional	444 (54.01)	3974 (94.13)	692 (81.22)
Non-Healthcare Professional	378 (45.99)	248 (5.87)	121 (14.2)
Not Specified	0 (0)	0 (0)	39 (4.58)
**EEA/Non-EEA**	822 (100)	4222 (100)	852 (100)
European Economic Area	229 (27.86)	865 (20.49)	340 (39.91)
Non-European Economic Area	593 (72.14)	3357 (79.51)	512 (60.09)
**Country**			
France	66 (8.03)	416 (9.85)	94 (11.03)
Germany	57 (6.93)	116 (2.75)	132 (15.49)
UK	16 (1.95)	101 (2.39)	0
Italy	28 (3.41)	64 (1.52)	24 (2.82)
Spain	30 (3.65)	50 (1.18)	17 (2)
Netherlands	0	32 (0.76)	0
Austria	0	15 (0.36)	0
Others	32 (3.89)	71 (1.68)	61 (7.16)
**Seriousness**	822 (100)	4222 (100)	852 (100)
Non-Serious	93 (11.3)	603 (14.3)	137 (16.1)
Serious	728 (88.6)	3619 (85.7)	713 (83.7)
Not Specified	1 (0.1)	0 (0)	2 (0.2)
**Outcome**	2220 (100)	8823 (100)	2081 (100)
Fatal	42 (1.89)	503 (5.7)	67 (3.22)
Not Recovered/Not Resolved	138 (6.22)	1169 (13.25)	278 (13.36)
Not Specified	0 (0)	0 (0)	37 (1.78)
Recovered/Resolved	300 (13.51)	1423 (16.13)	320 (15.38)
Recovered/Resolved with Sequelae	6 (0.27)	16 (0.18)	19 (0.91)
Recovering/Resolving	134 (6.04)	455 (5.16)	93 (4.47)
Unknown	1600 (72.07)	5257 (59.58)	1267 (60.88)

**Table 2:** Individual Case Safety Reports (ICSRs) stratified by system organ class (SOC). The number of reports with at least one adverse drug reaction (ADR) related to the SOCs are reported. The sum of the events by SOC (%) is higher than the total number of reports, since a single ICSR could include ADRs related to more than one SOC.

SOC	Stiripentol (%)	Cannabidiol (%)	Fenfluramine (%)
Blood and lymphatic system disorders	26 (1.2)	47 (0.6)	15 (0.8)
Cardiac disorders	2 (0.1)	85 (1)	269 (14)
Congenital, familial, and genetic disorders	1 (0.0)	13 (0.2)	8 (0.4)
Ear and labyrinth disorders	1 (0.0)	24 (0.3)	5 (0.3)
Endocrine disorders	3 (0.1)	12 (0.1)	4 (0.2)
Eye disorders	5 (0.2)	59 (0.7)	11 (0.6)
Gastrointestinal disorders	103 (4.9)	550 (6.5)	76 (4)
General disorders and administration site conditions	313 (14.8)	1200 (14.2)	234 (12.2)
Hepatobiliary disorders	15 (0.7)	61 (0.7)	15 (0.8)
Immune system disorders	5 (0.2)	50 (0.6)	6 (0.3)
Infections and infestations	117 (5.5)	551 (6.5)	83 (4.3)
Injury, poisoning, and procedural complications	435 (20.6)	1232 (14.5)	140 (7.3)
Investigations	105 (5)	428 (5.1)	112 (5.8)
Metabolism and nutritional disorders	127 (6)	207 (2.4)	97 (5)
Musculoskeletal and connective tissue disorders	27 (1.3)	80 (0.9)	20 (1)
Neoplasms: benign, malignant, and unspecified	1 (0.0)	26 (0.3)	7 (0.4)
Nervous system disorders	563 (26.6)	2292 (27.1)	368 (19.1)
Pregnancy, puerperium, and perinatal conditions	1 (0.0)	10 (0.1)	3 (0.2)
Product issues	6 (0.3)	203 (2.4)	8 (0.4)
Psychiatric disorders	117 (5.5)	566 (6.7)	109 (5.7)
Renal and urinary disorders	16 (0.8)	68 (0.8)	19 (1)
Reproductive system and breast disorders	2 (0.1)	16 (0.2)	7 (0.4)
Respiratory, thoracic, and mediastinal disorders	26 (1.2)	249 (2.9)	181 (9.4)
Skin and subcutaneous tissue disorders	32 (1.5)	151 (1.8)	22 (1.1)
Social circumstances	3 (0.1)	49 (0.6)	5 (0.3)
Surgical and medical procedures	60 (2.8)	173 (2)	36 (1.9)
Vascular disorders	4 (0.2)	70 (0.8)	62 (3.2)

**Table 3:** Reported suspected reactions included in the group “Nervous system disorders”. Reactions with less than 10 cases have been excluded.

Suspected Reaction	Stiripentol	Cannabidiol	Fenfluramine
Amnesia	/	11	/
Ataxia	11	10	/
Atonic seizures	/	19	/
Balance disorder	14	33	/
Cognitive disorder	/	15	/
Coma	/	15	/
Depressed level of consciousness	/	14	/
Disturbance in attention	/	15	/
Dizziness	/	42	22
Drooling	/	11	/
Dysarthria	/	12	/
Dyskinesia	/	11	/
Epilepsy	17	61	11
Generalized tonic–clonic seizure	14	79	15
Headache	/	38	14
Hypersomnia	/	25	/
Hypotonia	14	10	/
Lethargy	16	60	/
Loss of consciousness	/	17	/
Memory impairment	/	14	/
Myoclonic epilepsy	/	12	/
Partial seizures	/	12	/
Petit mal epilepsy	/	24	/
Psychomotor hyperactivity	/	11	/
Sedation	/	47	/
Seizure	423	1609	151
Seizure cluster	/	47	/
Somnolence	84	207	32
Speech disorder	/	10	/
Status epilepticus	10	60	23
Syncope	/	/	11
Tonic convulsion	/	14	/
Tremor	10	26	/

**Table 4:** Signal strength at the system organ class (SOC) level. ROR = reporting odds ratio.

SOC	ROR		
	Cannabidiol vs. Others	Fenfluramine vs. Others	Stiripentol vs. Others
Blood and lymphatic system disorders	0.448 [0.409; 0.491]	1.22 [1.04; 1.432]	2.64 [2.366; 2.947]
Cardiac disorders	0.106 [0.103; 0.11]	26.29 [25.422; 27.187]	0.033 [0.012; 0.087]
Congenital, familial, and genetic disorders	0.571 [0.395; 0.827]	3.406 [2.311; 5.018]	0.293 [0.037; 2.291]
Ear and labyrinth disorders	1.589 [1.055; 2.395]	1.185 [0.738; 1.902]	0.212 [0.028; 1.614]
Endocrine disorders	0.679 [0.435; 1.059]	1.581 [0.848; 2.95]	1.158 [0.532; 2.523]
Eye disorders	1.469 [1.255; 1.719]	1.018 [0.824; 1.258]	0.437 [0.287; 0.668]
Gastrointestinal disorders	1.251 [1.231; 1.272]	0.659 [0.638; 0.68]	1.018 [0.992; 1.044]
General disorders and administration site conditions	0.818 [0.812; 0.824]	0.884 [0.872; 0.896]	1.561 [1.542; 1.58]
Hepatobiliary disorders	0.803 [0.728; 0.887]	1.171 [0.999; 1.374]	1.222 [1.042; 1.433]
Immune system disorders	1.812 [1.455; 2.256]	0.643 [0.447; 0.927]	0.548 [0.357; 0.843]
Infections and infestations	1.106 [1.089; 1.123]	0.707 [0.686; 0.728]	1.162 [1.136; 1.189]
Injury, poisoning, and procedural complications	0.788 [0.782; 0.793]	0.398 [0.391; 0.406]	3.033 [2.998; 3.068]
Investigations	0.757 [0.746; 0.769]	1.281 [1.25; 1.312]	1.23 [1.199; 1.261]
Metabolism and nutritional disorders	0.334 [0.327; 0.34]	1.812 [1.76; 1.865]	2.867 [2.796; 2.94]
Musculoskeletal and connective tissue disorders	0.669 [0.625; 0.716]	1.109 [0.985; 1.249]	1.689 [1.536; 1.858]
Neoplasms benign, malignant, and unspecified (incl cysts and polyps)	1.29 [0.935; 1.781]	1.539 [1.079; 2.196]	0.186 [0.025; 1.406]
Nervous system disorders	0.948 [0.942; 0.954]	0.583 [0.577; 0.589]	1.973 [1.948; 1.998]
Pregnancy, puerperium, and perinatal conditions	0.991 [0.498; 1.972]	1.617 [0.702; 3.723]	0.474 [0.057; 3.925]
Product issues	5.989 [5.148; 6.968]	0.219 [0.17; 0.284]	0.169 [0.121; 0.238]
Psychiatric disorders	0.992 [0.978; 1.006]	0.937 [0.915; 0.959]	1.082 [1.057; 1.107]
Renal and urinary disorders	0.767 [0.703; 0.836]	1.347 [1.184; 1.533]	1.138 [0.982; 1.319]
Reproductive system and breast disorders	0.704 [0.5; 0.991]	2.313 [1.564; 3.422]	0.536 [0.184; 1.558]
Respiratory, thoracic, and mediastinal disorders	0.444 [0.436; 0.453]	4.678 [4.579; 4.779]	0.353 [0.325; 0.383]
Skin and subcutaneous tissue disorders	1.113 [1.057; 1.171]	0.704 [0.635; 0.78]	1.148 [1.064; 1.237]
Social circumstances	2.445 [1.836; 3.257]	0.567 [0.368; 0.873]	0.341 [0.17; 0.681]
Surgical and medical procedures	0.702 [0.679; 0.726]	0.911 [0.853; 0.973]	1.833 [1.752; 1.917]
Vascular disorders	0.411 [0.387; 0.436]	5.271 [4.959; 5.602]	0.183 [0.11; 0.304]

**Table 5:** Suspected reactions reported for fenfluramine included in the groups “Cardiac disorders”, “Vascular disorders”, and “Respiratory, thoracic, and mediastinal disorders”. Reactions with less than 10 cases have been excluded.

Cardiac Disorders	N Overall	N from 2021
Aortic valve incompetence	79	2
Cardiac failure	11	/
Cardiac failure congestive	15	/
Cardiac valve disease	51	2
Cardiovascular disorder	60	/
Left atrial dilatation	10	/
Left ventricular hypertrophy	13	/
Mitral valve incompetence	86	5
Mitral valve stenosis	11	/
Palpitations	16	1
Right ventricular failure	11	/
Tachycardia	19	4
Tricuspid valve incompetence	44	4
**Respiratory, thoracic, and mediastinal disorders**		/
Cough	13	7
Dyspnoea	63	/
Pulmonary arterial hypertension	27	/
Pulmonary hypertension	62	3
**Vascular disorders**		/
Hypertension	19	3

**Table 6:** Suspected reactions reported for cannabidiol included in the group ‘Product issues”. Reactions with less than 10 cases have been excluded.

Product Issues	N
Liquid product physical issue	14
Product container issue	10
Product distribution issue	29
Product quality issue	12
Product supply issue	96

**Table 7:** Suspected reactions reported for stiripentol included in the groups ‘Injury, poisoning, and procedural complications”, “Metabolism and nutrition disorders”, and “Blood and lymphatic system disorders”. Reactions with less than 10 cases have been excluded.

Blood and Lymphatic System Disorders	N
Neutropenia	13
Thrombocytopenia	11
**Injury, poisoning, and procedural complications**	
Fall	31
Inappropriate schedule of product administration	13
Off label use	20
Product dose omission issue	99
Product preparation issue	104
Product use in unapproved indication	233
Product use issue	19
Wrong technique in product usage process	19
**Metabolism and nutritional disorders**	
Decreased appetite	92
Dehydration	18
Hyperammonaemia	12

**Table 8:** Two-by-two contingency table for signal detection.

	SOC of Interest	Other SOCs	Total
**Active ingredient 1**	a	b	a + b
**Active ingredient 2 + 3**	c	d	c + d
**Total**	a + c	b + d	N

**Table 9:** Thresholds of signal detection methods. BCPNN = Bayesian confidence propagation neural network; MGPS = multi-item gamma Poisson shrinker; PRR = proportional reporting ratio; ROR = reporting odds ratio.

Signal Detection Methods	Thresholds
ROR	Lower limit of 95% CI of ROR > 1 and a ≥ 3/5
PRR	Lower limit of 95% CI of PRR > 1 and a ≥ 3/5
BCPNN	Lower limit of 95% *CI* of IC > 0
MGPS	5th percentile of EBGM (EB05) ≥ 1.8/2 and EBGM ≥ 2.5

## Data Availability

All data supporting the findings of this study are included in the article and Supplementary Material, further inquiries can be directed to the corresponding author.

## Supplementary Materials

The following supporting information can be downloaded at: https://www.mdpi.com/article/10.3390/ph18121895/s1, Table S1: Signal strength at the system organ class (SOC) level. PRR proportional reporting ratio; Table S2: Signal strength at the system organ class (SOC) level. χ2 chi-squared; Table S3: Signal strength at the system organ class (SOC) level. BCPNN = Bayesian confidence propagation neural network; Table S4: Signal strength at the system organ class (SOC) level. MGPS = multi-item gamma Poisson shrinker; Table S5: Individual Case Safety Reports (ICSRs) stratified by system organ class (SOC) and reporting area for cannabidiol.

## Abbreviations

The following abbreviations are used in this manuscript:

5-HT	serotonin
σ1-Rs	sigma-1 receptors
ADRs	adverse drug reactions
AEs	adverse events
AEDs	antiepileptic drugs
BCPNN	Bayesian confidence propagation neural network
BTCS	bilateral tonic–clonic seizures
CLB	clobazam
DS	Dravet syndrome
EB05	5th percentile of EBGM
EMA	European Medicines Agency
ENT1	nucleoside transporter 1
GABA	γ-aminobutyric acid
ICSRs	Individual Case Safety Reports
LGS	Lennox–Gastaut syndrome
MedDRA	Medical Dictionary for Regulatory Activities
MGPS	multi-item gamma Poisson shrinker
PAH	pulmonary arterial hypertension
PRR	proportional reporting ratio
ROR	reporting odds ratio
SMEI	severe myoclonic epilepsy of infancy
SmPCs	summary of product characteristics
SOCs	system organ classes
THC	Δ9-tetrahydrocannabinol
TRPV1	transient receptor potential vanilloid 1
VHD	valvular heart disease
VPA	valproate
