# Supplementary material for: Safety of Drugs Used in Difficult-to-Treat Epileptic Syndromes: A Disproportionality Analysis Using the Eudravigilance Database
Source: Pharmaceuticals (Basel). 2025 Dec 16;18(12):1895. doi: 10.3390/ph18121895 (PMC12736221; doi:10.3390/ph18121895)
Supplement: Supplementary file 1 [file pharmaceuticals-18-01895-s001.zip › pharmaceuticals-4030954-supplementary.pdf]

Table S1. Signal strength at the System Organ Class (SOC) level. PRR proportional reporting ratio,

SOC	PRR		
	**Cannabidiol vs others**	**Fenfluramine vs others**	**Stiripentol vs others**
Blood and lymphatic system disorders	0.455 [0.3; 0.688]	1.216 [0.701; 2.11]	2.589 [1.647; 4.068]
Cardiac disorders	0.124 [0.098; 0.158]	18.305 [14.535; 23.052]	0.035 [0.009; 0.14]
Congenital, familial and genetic disorders	0.573 [0.245; 1.337]	3.383 [1.423; 8.04]	0.294 [0.04; 2.182]
Ear and labyrinth disorders	1.586 [0.649; 3.873]	1.184 [0.455; 3.084]	0.213 [0.029; 1.56]
Endocrine disorders	0.68 [0.268; 1.723]	1.579 [0.525; 4.745]	1.157 [0.338; 3.963]
Eye disorders	1.462 [0.844; 2.533]	1.018 [0.539; 1.921]	0.441 [0.178; 1.089]
Gastrointestinal disorders	1.218 [1.039; 1.428]	0.689 [0.55; 0.864]	1.016 [0.836; 1.234]
General disorders and administration site conditions	0.87 [0.8; 0.946]	0.916 [0.815; 1.029]	1.347 [1.222; 1.485]
Hepatobiliary disorders	0.806 [0.523; 1.244]	1.168 [0.675; 2.023]	1.218 [0.704; 2.109]
Immune system disorders	1.802 [0.941; 3.453]	0.646 [0.279; 1.495]	0.551 [0.221; 1.372]
Infections and infestations	1.092 [0.939; 1.271]	0.736 [0.593; 0.913]	1.139 [0.949; 1.368]
Injury, poisoning and procedural complications	0.85 [0.783; 0.921]	0.497 [0.425; 0.581]	1.957 [1.809; 2.117]
Investigations	0.782 [0.671; 0.912]	1.244 [1.028; 1.505]	1.2 [0.987; 1.46]
Metabolism and nutrition disorders	0.366 [0.306; 0.439]	1.719 [1.388; 2.13]	2.579 [2.125; 3.129]
Musculoskeletal and connective tissue disorders	0.675 [0.473; 0.963]	1.107 [0.69; 1.774]	1.667 [1.097; 2.533]
Neoplasms benign, malignant and unspecified (incl cysts and polyps)	1.289 [0.585; 2.84]	1.535 [0.671; 3.513]	0.187 [0.026; 1.366]
Nervous system disorders	0.976 [0.928; 1.027]	0.763 [0.704; 0.827]	1.306 [1.239; 1.378]
Pregnancy, puerperium and perinatal conditions	0.991 [0.311; 3.156]	1.615 [0.451; 5.776]	0.475 [0.062; 3.625]
Product issues	5.749 [3.355; 9.852]	0.227 [0.112; 0.457]	0.176 [0.078; 0.394]
Psychiatric disorders	0.993 [0.86; 1.146]	0.945 [0.782; 1.141]	1.07 [0.892; 1.283]
Renal and urinary disorders	0.77 [0.514; 1.154]	1.339 [0.818; 2.192]	1.135 [0.67; 1.925]
Reproductive system and breast disorders	0.705 [0.312; 1.592]	2.302 [0.965; 5.496]	0.537 [0.127; 2.272]
Respiratory, thoracic and mediastinal disorders	0.477 [0.4; 0.568]	3.897 [3.278; 4.632]	0.373 [0.253; 0.551]
Skin and subcutaneous tissue disorders	1.109 [0.817; 1.505]	0.712 [0.46; 1.101]	1.142 [0.789; 1.653]

Social circumstances	2.429 [1.153; 5.117]	0.569 [0.228; 1.421]	0.343 [0.107; 1.094]
Surgical and medical procedures	0.715 [0.56; 0.911]	0.915 [0.649; 1.29]	1.772 [1.343; 2.339]
Vascular disorders	0.421 [0.302; 0.586]	4.96 [3.568; 6.896]	0.187 [0.069; 0.504]

Table S2. Signal strength at the System Organ Class (SOC) level. $\chi 2$ chi-squared

SOC	$\chi 2$		
	**Cannabidiol vs others**	**Fenfluramine vs others**	**Stiripentol vs others**
Blood and lymphatic system disorders	13,658	0,297	16,833
Cardiac disorders	422,074	1139,312	55,351
Congenital, familial and genetic disorders	1,140	6,890	0,934
Ear and labyrinth disorders	0,671	0,007	2,009
Endocrine disorders	0,317	0,243	0,010
Eye disorders	1,527	0,012	2,765
Gastrointestinal disorders	5,813	10,534	0,010
General disorders and administration site conditions	10,199	2,120	32,222
Hepatobiliary disorders	0,737	0,165	0,306
Immune system disorders	2,759	0,718	1,246
Infections and infestations	1,215	7,729	1,770
Injury, poisoning and procedural complications	14,823	93,912	221,694
Investigations	9,535	4,713	3,083
Metabolism and nutrition disorders	125,919	23,709	92,017
Musculoskeletal and connective tissue disorders	4,316	0,086	5,187
Neoplasms benign, malignant and unspecified (incl cysts and polyps)	0,194	0,603	2,589
Nervous system disorders	0,801	52,345	73,043
Pregnancy, puerperium and perinatal conditions	0,079	0,132	0,122
Product issues	52,229	20,220	22,499
Psychiatric disorders	0,003	0,289	0,450
Renal and urinary disorders	1,343	1,045	0,107
Reproductive system and breast disorders	0,388	2,709	0,325
Respiratory, thoracic and mediastinal disorders	69,371	252,527	27,229
Skin and subcutaneous tissue disorders	0,341	2,074	0,359

Social circumstances	5,144	1,073	2,920
Surgical and medical procedures	7,008	0,177	15,709
Vascular disorders	26,762	106,618	13,118

Table S3. Signal strength at the System Organ Class (SOC) level. BCPNN = Bayesian confidence propagation neural network

SOC	BCPNN		
	**Cannabidiol vs others**	**Fenfluramine vs others**	**Stiripentol vs others**
Blood and lymphatic system disorders	-0.431 [-0.435; -0.427]	0.206 [0.17; 0.242]	1.009 [0.996; 1.022]
Cardiac disorders	-1.577 [-1.578; -1.576]	2.359 [2.359; 2.36]	-4.082 [-5.056; -3.108]
Congenital, familial and genetic disorders	-0.319 [-0.363; -0.274]	1.057 [0.946; 1.168]	-1.073 [-3.257; 1.111]
Ear and labyrinth disorders	0.109 [0.095; 0.122]	0.13 [-0.116; 0.377]	-1.413 [-3.596; 0.771]
Endocrine disorders	-0.236 [-0.288; -0.185]	0.362 [0.008; 0.715]	0.078 [-0.473; 0.629]
Eye disorders	0.115 [0.113; 0.118]	0.001 [-0.062; 0.065]	-0.951 [-1.198; -0.705]
Gastrointestinal disorders	0.074 [0.074; 0.074]	-0.468 [-0.47; -0.467]	0.016 [0.015; 0.017]
General disorders and administration site conditions	-0.06 [-0.06; -0.06]	-0.111 [-0.111; -0.11]	0.359 [0.359; 0.359]
Hepatobiliary disorders	-0.109 [-0.111; -0.106]	0.162 [0.126; 0.198]	0.21 [0.174; 0.246]
Immune system disorders	0.169 [0.165; 0.172]	-0.509 [-0.691; -0.328]	-0.685 [-0.932; -0.439]
Infections and infestations	0.034 [0.034; 0.034]	-0.385 [-0.387; -0.384]	0.156 [0.155; 0.157]
Injury, poisoning and procedural complications	-0.071 [-0.071; -0.071]	-0.896 [-0.897; -0.895]	0.784 [0.784; 0.784]
Investigations	-0.111 [-0.111; -0.111]	0.26 [0.259; 0.261]	0.218 [0.217; 0.219]
Metabolism and nutrition disorders	-0.577 [-0.577; -0.576]	0.627 [0.626; 0.628]	1.063 [1.062; 1.064]
Musculoskeletal and connective tissue disorders	-0.193 [-0.195; -0.192]	0.106 [0.085; 0.128]	0.57 [0.558; 0.583]
Neoplasms benign, malignant and unspecified (incl cysts and polyps)	0.052 [0.04; 0.064]	0.401 [0.261; 0.54]	-1.556 [-3.74; 0.627]
Nervous system disorders	-0.01 [-0.01; -0.01]	-0.34 [-0.34; -0.34]	0.323 [0.323; 0.324]
Pregnancy, puerperium and perinatal conditions	-0.093 [-0.165; -0.022]	0.336 [-0.214; 0.886]	-0.629 [-2.813; 1.555]
Product issues	0.377 [0.377; 0.378]	-1.853 [-1.964; -1.742]	-2.166 [-2.348; -1.984]
Psychiatric disorders	-0.004 [-0.004; -0.004]	-0.072 [-0.073; -0.071]	0.08 [0.079; 0.081]
Renal and urinary disorders	-0.129 [-0.131; -0.127]	0.319 [0.295; 0.342]	0.133 [0.1; 0.165]
Reproductive system and breast disorders	-0.206 [-0.236; -0.176]	0.749 [0.61; 0.889]	-0.625 [-1.6; 0.35]
Respiratory, thoracic and mediastinal disorders	-0.392 [-0.392; -0.392]	1.44 [1.44; 1.441]	-1.262 [-1.275; -1.249]
Skin and subcutaneous tissue disorders	0.034 [0.034; 0.035]	-0.42 [-0.438; -0.403]	0.15 [0.141; 0.159]

Social circumstances	0.234 [0.23; 0.237]	-0.645 [-0.892; -0.399]	-1.184 [-1.735; -0.634]
Surgical and medical procedures	-0.159 [-0.159; -0.159]	-0.114 [-0.121; -0.106]	0.658 [0.655; 0.661]
Vascular disorders	-0.48 [-0.482; -0.479]	1.598 [1.596; 1.601]	-2.008 [-2.362; -1.654]

Table S4. Signal strength at the System Organ Class (SOC) level. MGPS = multi-item gamma Poisson shrinker

SOC	MGPS		
	**Cannabidiol vs others**	**Fenfluramine vs others**	**Stiripentol vs others**
Blood and lymphatic system disorders	0.746 [0.489; 1.138]	1.18 [0.673; 2.066]	2.119 [1.332; 3.371]
Cardiac disorders	0.333 [0.259; 0.429]	5.229 [4.046; 6.758]	0.04 [0.01; 0.162]
Congenital, familial and genetic disorders	0.825 [0.352; 1.934]	2.516 [1.052; 6.017]	0.326 [0.044; 2.427]
Ear and labyrinth disorders	1.117 [0.456; 2.738]	1.153 [0.44; 3.021]	0.239 [0.033; 1.758]
Endocrine disorders	0.882 [0.347; 2.244]	1.457 [0.482; 4.4]	1.133 [0.329; 3.895]
Eye disorders	1.099 [0.63; 1.914]	1.015 [0.533; 1.932]	0.478 [0.192; 1.188]
Gastrointestinal disorders	1.054 [0.881; 1.26]	0.721 [0.562; 0.926]	1.013 [0.811; 1.266]
General disorders and administration site conditions	0.959 [0.849; 1.084]	0.927 [0.788; 1.09]	1.285 [1.102; 1.498]
Hepatobiliary disorders	0.936 [0.602; 1.455]	1.141 [0.652; 1.994]	1.182 [0.676; 2.067]
Immune system disorders	1.145 [0.594; 2.204]	0.681 [0.292; 1.586]	0.588 [0.235; 1.472]
Infections and infestations	1.025 [0.862; 1.218]	0.765 [0.601; 0.973]	1.117 [0.903; 1.382]
Injury, poisoning and procedural complications	0.952 [0.844; 1.074]	0.536 [0.443; 0.649]	1.727 [1.486; 2.007]
Investigations	0.927 [0.779; 1.103]	1.202 [0.966; 1.494]	1.168 [0.934; 1.46]
Metabolism and nutrition disorders	0.671 [0.55; 0.818]	1.557 [1.227; 1.977]	2.114 [1.693; 2.639]
Musculoskeletal and connective tissue disorders	0.88 [0.611; 1.267]	1.09 [0.672; 1.767]	1.525 [0.99; 2.348]
Neoplasms benign, malignant and unspecified (incl cysts and polyps)	1.068 [0.483; 2.364]	1.425 [0.618; 3.282]	0.211 [0.029; 1.545]
Nervous system disorders	0.993 [0.886; 1.113]	0.79 [0.682; 0.915]	1.253 [1.071; 1.466]
Pregnancy, puerperium and perinatal conditions	0.997 [0.312; 3.185]	1.483 [0.413; 5.326]	0.512 [0.067; 3.922]
Product issues	1.306 [0.758; 2.252]	0.255 [0.125; 0.519]	0.198 [0.088; 0.448]
Psychiatric disorders	0.998 [0.846; 1.178]	0.952 [0.767; 1.183]	1.06 [0.857; 1.31]
Renal and urinary disorders	0.922 [0.611; 1.392]	1.277 [0.772; 2.112]	1.114 [0.65; 1.909]
Reproductive system and breast disorders	0.894 [0.394; 2.027]	1.938 [0.807; 4.653]	0.574 [0.135; 2.438]
Respiratory, thoracic and mediastinal disorders	0.763 [0.628; 0.926]	2.747 [2.239; 3.369]	0.409 [0.273; 0.612]
Skin and subcutaneous tissue disorders	1.029 [0.75; 1.411]	0.743 [0.474; 1.163]	1.12 [0.762; 1.645]

Social circumstances	1.2 [0.567; 2.54]	0.607 [0.242; 1.524]	0.378 [0.118; 1.21]
Surgical and medical procedures	0.898 [0.695; 1.16]	0.926 [0.647; 1.326]	1.6 [1.189; 2.153]
Vascular disorders	0.719 [0.511; 1.011]	3.155 [2.233; 4.458]	0.211 [0.078; 0.572]

Table S5. Individual Case Safety Reports (ICSRs) stratified by system organ class (SOC) and reporting area for cannabidiol. EEA = European Economic Area; NS = not specified

SOC	EEA (%)	Non EEA (%)	Total
Blood and lymphatic system disorders	22 (46.8)	25 (53.2)	47
Cardiac disorders	31 (36.5)	54 (63.5)	85
Congenital, familial and genetic disorders	0	13 (100)	13
Ear and labyrinth disorders	16 (66.7)	8 (33.3)	24
Endocrine disorders	2 (16.7)	10 (83.3)	12
Eye disorders	24 (40.7)	35 (59.3)	59
Gastrointestinal disorders	159 (28.9)	391 (71.1)	550
General disorders and administration site conditions	257 (21.4)	943 (78.6)	1,200
Hepatobiliary disorders	33 (54.1)	28 (45.9)	61
Immune system disorders	12 (24)	38 (76)	50
Infections and infestations	32 (5.8)	519 (94.2)	551
Injury, poisoning and procedural complications	125 (10.1)	1,107 (89.9)	1,232
Investigations	114 (26.6)	314 (73.4)	428
Metabolism and nutrition disorders	68 (32.9)	139 (67.1)	207
Musculoskeletal and connective tissue disorders	22 (27.5)	58 (72.5)	80
Neoplasms benign, malignant and unspecified (incl cysts and polyps)	0	26 (100)	26
Nervous system disorders	326 (14.2)	1,966 (85.8)	2,292
Pregnancy, puerperium and perinatal conditions	2 (20)	8 (80)	10
Product issues	29 (14.3)	174 (85.7)	203
Psychiatric disorders	259 (45.8)	307 (54.2)	566
Renal and urinary disorders	12 (17.6)	56 (82.4)	68
Reproductive system and breast disorders	4 (25)	12 (75)	16
Respiratory, thoracic and mediastinal disorders	30 (12)	219 (88)	249
Skin and subcutaneous tissue disorders	62 (41.1)	89 (58.9)	151
Social circumstances	13 (26.5)	36 (73.5)	49
Surgical and medical procedures	4 (2.3)	169 (97.7)	173
Vascular disorders	25 (35.7)	45 (64.3)	70
**Total**	865 (20.5)	3,357 (79.5)	4,222
